# Acute Onset of Heart Failure and Renal Failure Due to Pacemaker Syndrome

**DOI:** 10.7759/cureus.37685

**Published:** 2023-04-17

**Authors:** Afreen Ashraf, Adrian G Dumitrascu, Kaitlin M Moran, Razvan M Chirila, Michael Smerina

**Affiliations:** 1 Hospital Medicine, Mayo Clinic, Jacksonville, USA; 2 Internal Medicine, Mayo Clinic, Jacksonville, USA

**Keywords:** pacemaker, heart block, complete heart block, pacemaker complication, tricuspid regurgitation, heart failure with reduced ejection fraction, pacemaker syndrome

## Abstract

Here, we report the outcome of an 87-year-old man with permanent non-valvular atrial fibrillation who initially presented with complete heart block and received a single right ventricle lead pacemaker programmed to ventricular demand pacing (VVIR). Over the next 10 months, the patient was readmitted to the hospital four times with recurrent edema, pleural effusions, and ascites. He was diagnosed with new onset systolic heart failure with mid-range (40-49%) ejection fraction and cardiorenal syndrome requiring dialysis. The underlying cause of his presentation was determined to be pacemaker syndrome mediated by new onset severe tricuspid regurgitation. He was treated with reimplantation of a pacemaker with His bundle pacing with subsequent improvement in his cardiac status and renal function. Implantation of dual-chamber pacing* *(DDDR) or His bundle pacing to achieve a narrow QRS complex over ventricular demand pacemaker is recommended whenever possible to reduce the incidence of pacemaker syndrome and improve patient outcomes.

## Introduction

Cardiac pacemakers are generally composed of a pulse generator generating the electrical impulse and one or more epicardially or endocardially placed leads, which deliver the electrical impulse from the generator to the myocardium. Leadless pacemakers are also newly available [1]. Cardiac pacemakers are the mainstay treatment for patients with non-reversible complete heart block (CHB). Other common indications for pacemaker placement are management of sinus node dysfunction, type II second-degree heart block, chronic bifascicular block, or neurocardiogenic syncope [2]. In a patient with atrial fibrillation (AFib) and CHB requiring permanent pacemaker (PPM) placement, the pacing is limited to single-ventricular or bi-ventricular. In the case of a patient without systolic heart failure, a bi-ventricular pacemaker would not be indicated commonly resulting in the placement of a ventricular demand pacing (VVIR) device (ventricle paced, ventricle sense, inhibited in response to a sensed beat) [3].

Choosing the ideal pacing mode for your patient may reduce the risk of adverse events and costs and will ultimately reduce the risk of pacemaker syndrome. VVIR has been identified as a common cause of pacemaker syndrome due to atrioventricular (AV) dyssynchrony and interventricular dyssynchrony [4,5]. It is estimated that upwards of one in five patients are at risk of developing pacemaker syndrome due to VVIR pacing [5]. The term “pacemaker syndrome” was introduced by Mitsui et al. in 1969 to describe symptoms attributed to AV dissociation secondary to VVIR pacing [6]. More broadly, the definition can be expanded to the adverse hemodynamics associated with a normally functioning pacing system resulting in overt symptoms or limitation of the patient’s ability to achieve optimal functional status [4]. Signs and symptoms of pacemaker syndrome include fatigue, dyspnea on exertion, orthopnea, paroxysmal nocturnal dyspnea, peripheral edema, increased jugular venous pressure, pre-syncope, and syncope. Clinicians should maintain a high index of suspicion for pacemaker syndrome in patients who develop signs and symptoms of heart failure (HF) after PPM placement. Here, we present a case of a patient who developed pacemaker syndrome after PPM placement for CHB.

## Case presentation

**Figure 1:**
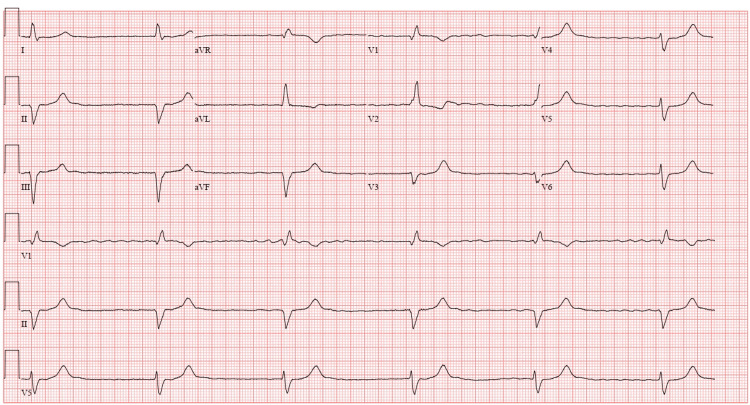
Initial ECG showing complete heart block with underlying atrial fibrillation, heart rate of 35 beats per minute, and QRS duration of 160 milliseconds.

**Figure 2:**
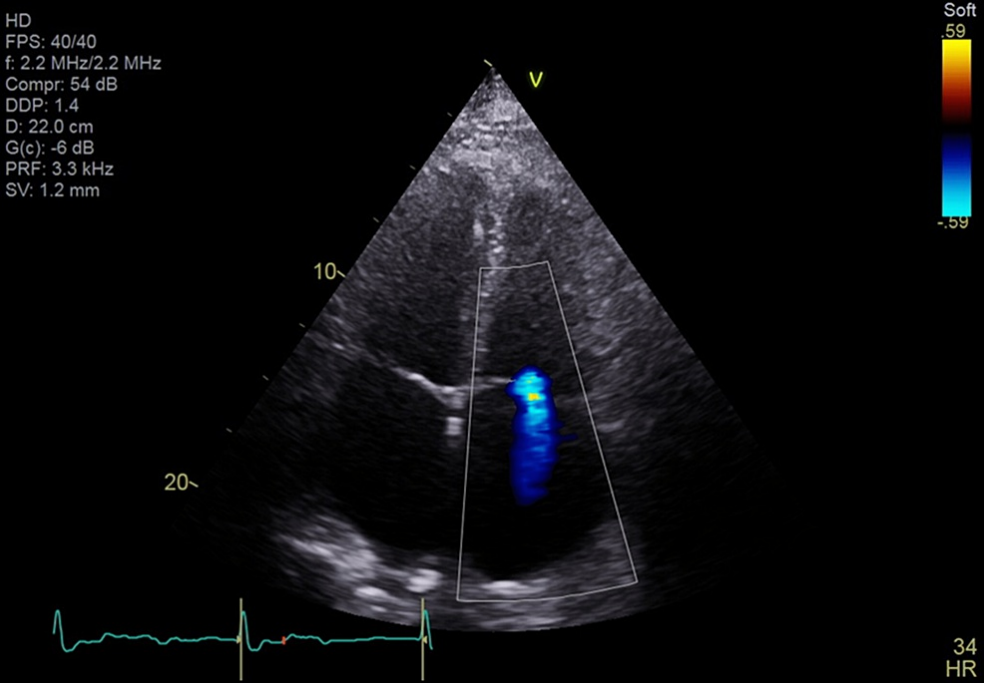
Baseline transthoracic echocardiogram of trivial tricuspid regurgitation prior to pacemaker placement. By Mayo Clinic convention, the left ventricle is inverted on the image (left upper cardiac chamber on image).

**Figure 3:**
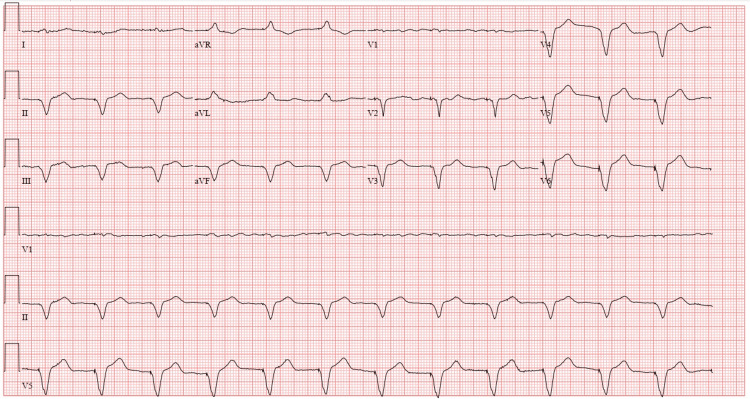
ECG status post placement of single lead permanent pacemaker showing ventricular paced rhythm at 70 beats per minute and QRS duration of 174 milliseconds.

An 87-year-old man with permanent non-valvular atrial fibrillation on anticoagulation, coronary artery disease status post bare metal stent placement to the left anterior descending artery in 2008 after non-ST-elevation myocardial infarction, and chronic kidney disease stage 3 presented to the hospital with dyspnea on exertion without anginal symptoms. On admission, his vitals at rest were a blood pressure of 120/50 millimeters of mercury (mmHg), a heart rate of 35 beats per minute (bpm), a respiratory rate of 18 per minute, a body weight of 93 kilograms (kg), and an oral temperature of 37°C. His laboratory data were significant for a hemoglobin of 12.8 grams/deciliter (g/dL), a thyroid stimulating hormone level of 2.4 microunits per milliliter, initial fifth-generation serum troponins at 26 nanograms per liter (ng/L) with a two-hour delta of zero, and serum creatinine of 1.5 milligrams per deciliter (mg/dL), which was his baseline. He was assessed with an electrocardiogram (ECG) and was found to have new onset CHB (Figure 1). The patient underwent a transthoracic echocardiogram (TTE), which showed a normal right ventricular systolic pressure (RVSP), mild bi-atrial enlargement, no wall motion abnormalities with a calculated left ventricular ejection fraction (EF) of 56%, mild mitral regurgitation (MR), and mild tricuspid regurgitation (TR) (Figure 2). He received no intravenous fluids and his oral AV nodal blocker was held without resolution of the CHB. His workup did not reveal a reversible etiology, and a single right ventricle (RV) lead PPM was implanted (Figure 3). The PPM was programmed to VVIR at a rate of 70 bpm.

**Figure 4:**
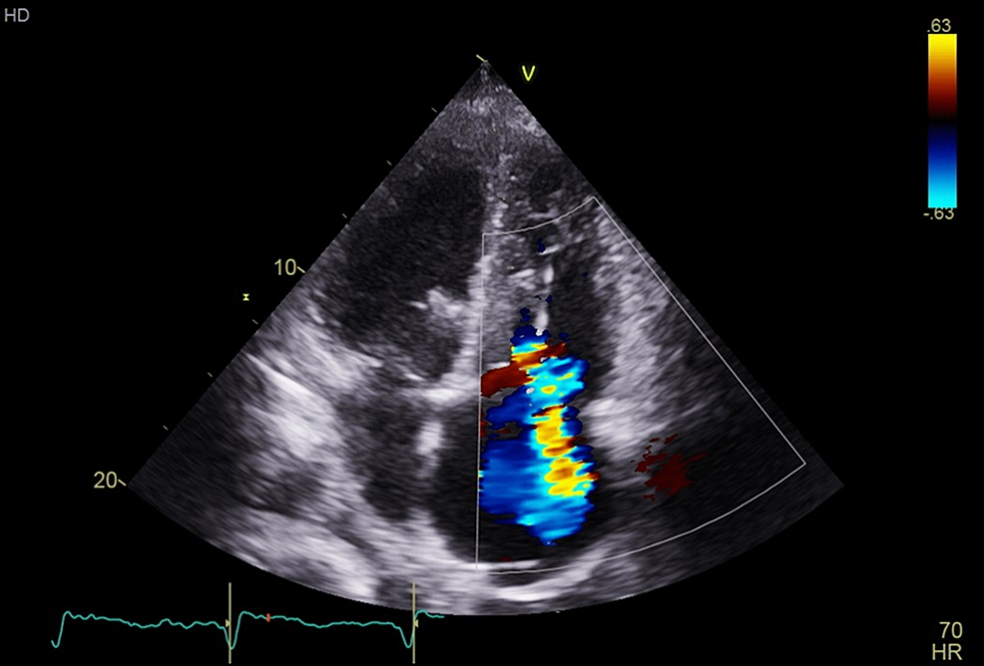
Repeat transthoracic echocardiogram at 10 months showing pacemaker lead and interval development of severe tricuspid regurgitation. By Mayo Clinic convention, the left ventricle is inverted on the image (left upper cardiac chamber on image).

Over the next 10 months, the patient was readmitted to the hospital four times with acute decompensated heart failure manifested as diuretic refractory volume overload and progressive renal failure. On his fourth visit, his admission vitals at rest were a blood pressure of 111/58 mmHg, a heart rate of 70 bpm, a respiratory rate of 18 per minute, a body weight of 108.4 kg, and an oral temperature of 37°C. Initial laboratory data were significant for a hemoglobin of 12.2 g/dL, first-fifth generation serum troponins at 47 ng/L with a two-hour delta of negative two, and serum creatinine of 3.5 mg/dL. Physical exam was consistent with anasarca, bilateral pleural effusions, and ascites. A repeat TTE showed moderate-severe TR, mild-moderate MR, moderate-severe bi-atrial enlargement, moderately reduced RV systolic function, increased RVSP, abnormal septal motion due to pacing, and a calculated left ventricular EF of 45% (Figure 4). The TTE was not suggestive of infiltrative disorders such as amyloidosis or sarcoidosis. During his admission, blood cultures remained negative. Interrogation of the PPM confirmed VVIR settings with 99.05% RV pacing with one episode of non-sustained ventricular tachycardia. The patient's QRS duration was 174 ms after PPM placement.

**Figure 5:**
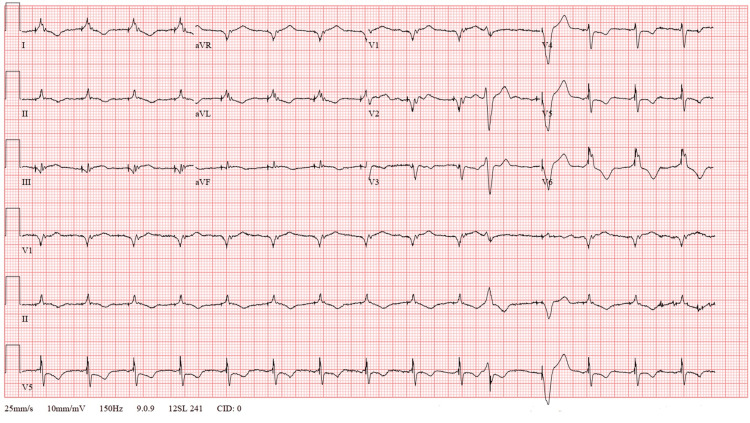
ECG showing improvement in ventricular paced rhythm at 90 beats per minute and QRS duration of 126 milliseconds.

**Figure 6:**
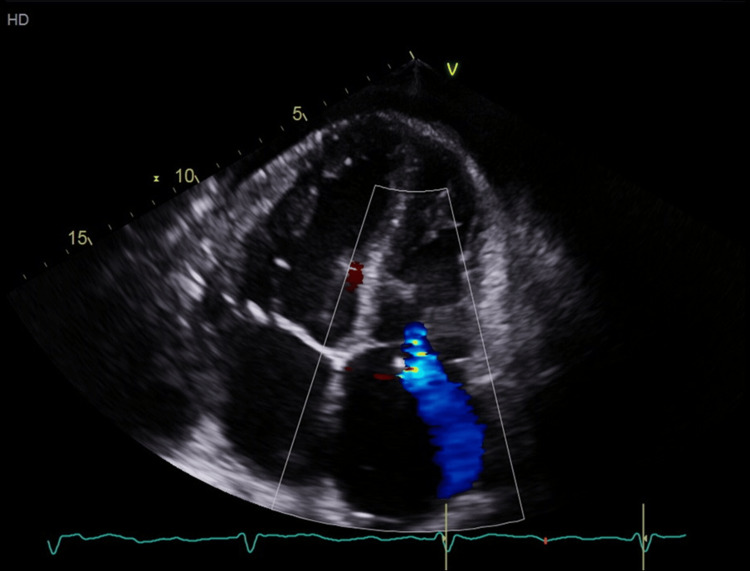
Transthoracic echocardiogram eight months after pacemaker upgrade and 18 months from initial pacemaker placement showing a reduction in tricuspid regurgitation to a grade of mild to moderate. By Mayo Clinic convention, the left ventricle is inverted on the image (left upper cardiac chamber on image).

He was initiated on intensive inpatient intravenous diuretic therapy for two weeks. Despite an 11 kg weight loss attributed to diuresis, his serum creatinine remained above 4 mg/dL and he was initiated on hemodialysis (HD) to optimize his volume status and discharged with close follow-up. Three months after initiating dialysis and maintaining his dry weight of 93 kg, he remained oliguric and dependent on dialysis. Due to his age, lack of delta in his serum troponins, and renal status, shared decision-making was used and he did not undergo a left heart catheterization. His symptoms and physical examination were not suggestive of carcinoid syndrome. The patient’s newly developed systolic left heart failure with mid-range EF, severe TR, and moderate right heart failure associated with progressive renal failure was suspected to be secondary to pacemaker syndrome. He underwent a PPM upgrade to a cardiac resynchronization therapy pacemaker. Multiple attempts to place a lead in the coronary sinus for left ventricular (LV) pacing were unsuccessful due to the severe TR; however, a His bundle lead was successfully placed. The patient’s QRS complex narrowed from 174 ms to 126 ms (Figure 5). The patient’s new PPM was programmed to dual-chamber pacing (DDDR) at 80 to 120 beats per minute. Repeat TTE after PPM replacement noted significant improvement in the patient's TR (Figure 6). The repeat TTE also ruled out mechanical interference of the pacing lead as a factor contributing to the patient's TR. Approximately three months later, the patient's renal function returned to his prior baseline without further requirement of HD. Since the pacemaker upgrade, the patient had not been readmitted for acute decompensated heart failure or volume overload strongly suggesting pacemaker syndrome as the root cause of his new onset systolic heart failure with mid-range EF and cardiorenal syndrome.

## Discussion

There exists a strong correlation between the burden of lone ventricular pacing and pacemaker syndrome. Link et al.’s Mode Selection Trial (MOST) trial found that 18.3% of patients treated with VVIR pacing for sick sinus syndrome develop pacemaker syndrome when compared to rate-adaptive atrioventricular synchronous dual-chamber pacing (DDDR) [5]. This case highlights the potential for adverse hemodynamic effects of pacemaker implantation with a high RV pacing burden and wide QRS. Patients who develop pacemaker syndrome have symptoms of low cardiac output, which may be as pronounced as the development of systolic heart failure, hypotension, and cardiogenic shock or more subtle, including dizziness, fatigue, cough, and dyspnea [4]. Although this diagnosis may be straightforward if symptoms appear shortly following the PPM implantation, sometimes prior comorbidities and advanced age coupled with an insidious presentation may confound the picture. In many cases, the pacemaker syndrome may be a clinical diagnosis of exclusion and providers must maintain a high index of suspicion after pacemaker placement for those who develop these signs and symptoms, as in the case presented. Additionally, in patients with permanent AFib and CHB treated only with a single chamber pacemaker in VVIR mode and having a high proportion of paced cardiac activity, the predominance of a widened QRS with pacing may be a marker for the risk of development of pacemaker syndrome. In the case presented, the RV pacing leading to the widened QRS contributed to interventricular dyssynchrony and valvular dysfunction. This combination was the proposed cause of the decreased cardiac output. This proposed mechanism is supported by the subsequent improvement in valvular function and symptoms after cardiac resynchronization with the placement of pacemaker wires to provide His bundle pacing thereby narrowing the QRS.

Classically, pacemaker syndrome was defined as the development of the above-described signs and symptoms due to atrioventricular dyssynchrony. LV-RV dyssynchrony, valvular incompetence, and inappropriate circulatory reflexes due to pacing have also been described as major contributors to the development of pacemaker syndrome [4,7,8]. In the case presented, the patient’s pacemaker syndrome was thought to be significantly mediated by the development of severe tricuspid regurgitation in the presence of AFib and CHB, as previously described [9]. Mechanical interference of the pacing lead sometimes can cause TR, but this was not the case in our patient, as a follow-up TTE showed resolution of TR after PPM upgrade and reduction in QRS duration. Although right ventricular demand pacing is a lower cost and technically easier approach to treating CHB, there is support for the up-front placement of dual-chamber pacemakers for patients with CHB with the goal of preventing the development of heart failure, valvular regurgitation, and the prospect of improving functional capacity and ultimately the quality of life [10-13].

## Conclusions

Despite having a normal baseline ejection fraction, normal baseline valvular function, and near-normal renal function, our patient’s clinical condition worsened rapidly and inexplicably. The underlying cause for the patient’s deterioration was not readily found, despite his recurrent admissions for heart failure exacerbation. The case presented above highlights the unpredictable and insidious development of this condition - the pacemaker syndrome. Providers must maintain a high index of suspicion in patients who develop new symptoms of decreased cardiac output after pacemaker implantation as they may be due to pacemaker syndrome. With corrective action, this condition may be reversible with subsequent improvement in a patient’s symptoms, quality of life, and organ function. Additionally, a focus on minimizing QRS duration in pacemaker-dependent patients may reduce the occurrence of pacemaker syndrome, especially in the case of both permanent AFib and CHB. In cases where cardiac resynchronization is required, and coronary venous lead cannot be readily implanted, His bundle pacing may show significant benefit.
